# SCONe: a community-acquired retinal image repository enabling ocular, cardiovascular and neurodegenerative disease prediction

**DOI:** 10.1136/bmjhci-2024-101236

**Published:** 2025-05-14

**Authors:** Claire Tochel, Miguel O Bernabeu, Alice McTrusty, Andrew J Tatham, Emma Pead, Fiona Buckmaster, Jonathan Penny, Tom MacGillivray, Malihe Javidi, Heather Anderson, Ana Paula Rubio, Robert Wallace, Jamie B R Kidd, Ruairidh MacLeod, Niall Strang, Baljean Dhillon

**Affiliations:** 1 Centre for Medical Informatics, The University of Edinburgh Usher Institute, Edinburgh, UK; 2 The University of Edinburgh Centre for Clinical Brain Sciences, Edinburgh, UK; 3 Princess Alexandra Eye Pavilion, NHS Lothian, Edinburgh, UK; 4 Royal College of Surgeons of Edinburgh, Edinburgh, UK; 5 Robert O Curle Ophthalmology Suite, Institute for Regeneration and Repair, The University of Edinburgh, Edinburgh, UK; 6 Public Health Scotland, Edinburgh, UK; 7 EPCC, The University of Edinburgh, Edinburgh, UK; 8 Life Sciences, Glasgow Caledonian University, Glasgow, UK; 9 Ophthalmology, Princess Alexandra Eye Pavilion, Edinburgh, UK

**Keywords:** Data Science, Electronic Health Records, Medical Record Linkage, Health Services Research, Medical Informatics

## Abstract

**Objectives:**

To safeguard Scotland’s community-acquired retinal images (colour fundus photographs) in a secure, centrally held repository and support a variety of research including ocular, neurodegenerative and systemic disease prediction.

**Design:**

Retinal images captured in optometry practices linked to national, routinely collected, longitudinal healthcare data.

**Setting:**

Community optometry and the Public Health Scotland National Safe Haven.

**Participants:**

Adults (mostly aged 60+) who have attended their optometrist since 2006 for an eye examination during which a retinal image was captured.

**Main outcome measures:**

Successful retrieval of linkable colour fundus photographs from systems in use in practice and delivery to the Safe Haven for linkage and secure storage.

**Results:**

Scottish Collaborative Optometry-Ophthalmology Network e-research (SCONe) currently contains over 367 000 retinal images matched to over 36 000 patients. Healthcare data (hospital inpatient and outpatient, general ophthalmic, death and prescribing) records were retrieved for patients with one or more images, providing demographic and healthcare information for 95% of the cohort. The linked data allow the application of condition labels or phenotypes at specific points in time, facilitating research into retinal manifestations of vascular and neural diseases. The cohort is representative of the Scottish 60+ population in terms of sex (54% female), and there is a slight over-representation of people of black, Asian and minority ethnic groups (2% vs 1%) and those living in areas of lower deprivation (30% vs 16% in lowest two categories). Early research work has begun and is focusing on ocular and neurodegenerative disease prediction.

**Conclusions:**

The SCONe retinal image repository has been successfully established. We believe it offers enormous potential to support research into earlier detection of disease.

**WHAT IS ALREADY KNOWN ON THIS TOPIC**

- Retinal images are known to contain information about ocular, systemic and neurodegenerative pathology before the development of symptoms. Retinal images captured during routine optometric examinations represent a, so far untapped, invaluable source of health evidence.

**WHAT THIS STUDY ADDS**

- This paper describes our creation of a large community-acquired retinal image repository, the linked data it contains and the representativeness of the cohort.

**HOW THIS STUDY MIGHT AFFECT RESEARCH, PRACTICE OR POLICY**

- This resource is now supporting research into disease prediction and offers the potential to support the development of tools which could facilitate earlier diagnoses and the identification of patients at highest risk of rapid progression.

## Introduction

 The retina is a uniquely accessible portal permitting direct observation of the neurovascular tissue bed in vivo through non-invasive, high-resolution, multimodal digital imaging.^1^ Harnessing this retinal readout as a platform for disease prediction is coming of age through the rapid evolution of artificial intelligence (AI)-enabled computational tools and technologies. However, realising the potential of this novel pathway to disease stratification and prediction modelling relies on robust validation through longitudinal, representative, curated, linked datasets which are currently lacking. Many diseases—ocular, systemic and neurodegenerative—are associated with changes in the retina.^2^,^4^ As retinal signs may manifest years before the emergence of symptoms, retinal imaging provides a prospective means for earlier disease detection.^5^,^7^ Retinal changes may also be an important prognostic sign which could improve risk stratification and disease trajectory prediction.

The Scottish Collaborative Optometry-Ophthalmology Network e-research (SCONe) retinal image repository (https://www.ed.ac.uk/clinical-sciences/ophthalmology/scone) is a big data resource comprising more than 15 years of community-acquired retinal images securely held within the Scottish National Safe Haven, a Trusted Research Environment owned by Public Health Scotland (PHS) (https://publichealthscotland.scot/services/data-research-and-innovation-services/electronic-data-research-and-innovation-service-edris/national-safe-haven-nsh/). These digital colour fundus photographs, captured during regular eye examinations in optometry practices, represent an unprecedented resource containing large numbers of images from healthy and diseased individuals. Images are linked (via the Community Health Index (CHI), the unique patient identifier used in Scotland) to longitudinal, coded, national health data facilitating the assignment of diagnostic labels at specific time points.

There remains a pressing need for improved identification of vision-threatening eye diseases such as age-related macular degeneration (AMD) and glaucoma, for which earlier diagnosis and treatment is associated with better long-term outcomes.^8^ In addition, retinal changes are a promising biomarker for neurodegenerative diseases such as Alzheimer’s disease (the leading cause of dementia globally).^9^ Multiple studies and meta-analyses have concluded that it is possible to develop automated approaches to image grading for the diagnosis of disease.^36 9^,^15^ However, the datasets used to develop such technologies are often not linked to patient outcomes or history or contain longitudinal images of disease progression. The SCONe repository offers the potential to support the development and evaluation of image analysis and risk prediction tools using retinal images from primary care, including those from asymptomatic individuals attending routine sight tests. The resource has been recognised in the Scottish Government Primary Care Directorate national guidance which described SCONe as ‘globally important’ with potential to have significant impact.^16^ Here we present the first description of the cohort after more than 3 years of work to create it and a discussion of the type of research it will be best placed to support.

## Methods

### Data resources

The SCONe cohort (as of April 2024) comprises patients with one or more retinal images from 14 participating independent community optometry practices in Scotland. We extracted images from practice systems with sufficient information (forename, surname and date of birth, sex, address and postcode) to allow PHS to link them robustly to each patient’s CHI. The uploaded images and associated metadata are processed by the Edinburgh Parallel Computing Company (EPCC) team (The University of Edinburgh) into a database for data management and to enable linkage. After linkage, the PHS electronic Data for Research & Innovation Service (eDRIS) team assigned a pseudonymous study ID, removed directly identifiable personal data and retrieved electronic healthcare records for each patient with one or more images. The pseudonymised SCONe repository therefore comprises fundus photographs, healthcare records and look-up files with robust linkage via a pseudonymous study ID. A schematic of this data flow is provided in the online supplemental material.

The repository includes hospital inpatient (Scottish Morbidity Record (SMR) 01), hospital outpatient (SMR00), General Ophthalmic Service (GOS1) and community-prescribed medication (Prescribing Information System (PIS)) records as well as diagnoses recorded in the National Records of Scotland Death Register. Each record is dated and contains a variety of contextual information about the patient or clinical encounter during which the code was recorded, such as demographics (eg, age, ethnic group, sex), socioeconomics (eg, deprivation using the Scottish Index of Multiple Deprivation (SIMD)), specialty (eg, general medicine, respiratory, ophthalmology), admission (eg, routine elective, urgent, emergency) and discharge route (eg, regular, transfer, death). The hospital datasets do not contain clinical information such as blood pressure or weight/height. The GOS1 records contain refraction but not other clinical information such as intraocular pressure. Prescription data include all community-dispensed items with strength, formulation, quantity and date. More detail on all PHS datasets is available online (https://publichealthscotland.scot/resources-and-tools/health-intelligence-and-data-management/national-data-catalogue/). Personally identifiable information is removed or provided with reduced specificity to create pseudonymous records which protect patient privacy but retain valuable information on confounding factors, for example, age at appointment date rather than date of birth. SIMD is based on the patient’s postcode at a given time, rather than an identifiable location. SIMD is a relative measure of deprivation for Scottish postcodes, which takes into account income, employment, education, health, access to services, crime and housing (https://publichealthscotland.scot/services/geography-population-and-deprivation-support/deprivation/scottish-index-of-multiple-deprivation-simd). Data sources were compiled to create a tidy demographic dataset which minimised missing data, as not all patients with a fundus photograph in the repository have a linked healthcare record from each source.

### Data Governance

The use of unconsented data within Safe Havens is approved by the Scottish Government who set out guidelines for this in their Charter for Safe Havens.^4^ Access is strictly limited to researchers at approved institutions, with appropriate governance training. Other groups must enter into partnerships with approved institutions to work with the data. No identifiable or potentially identifiable data can be removed from the Safe Haven—only aggregate research outputs, and only after a disclosure check by eDRIS, PHS. The creation of the SCONe retinal image repository and analysis of the data has been approved by the NHS Scotland Public Benefit and Privacy Panel for Health and Social Care (https://www.informationgovernance.scot.nhs.uk/pbpphsc/) at two stages of development: proof of concept (1920-0121, October 2021) and phase 2 (2223-0189, August 2023).

Before any images were copied, a Data Sharing Agreement was signed between the data controller in each participating practice and the study’s cosponsors: The University of Edinburgh and NHS Lothian. EPCC provides the National Safe Haven information technology infrastructure for PHS. It is a UK Statistics Authority Digital Economy Act Accredited Processing Environment, and EPCC holds certifications in ISO 27001 (Information Security) and IASME Cyber Essentials.

### Patient and public involvement and professional engagement

From its inception, SCONe was developed with input from patients involving representatives from organisations such as the Macular Society and Action Against Age-related Macular Degeneration. Most funding to date has come from patient-focused charities, in particular, Sight Scotland who represents groups affected by sight impairment. Input from project ambassadors, people already affected by visual loss who are committed to supporting research for the benefit of the next generation, helped shape the ethos of the project, and their stories are shared publicly on our website.^17^ Recently, we established a formal patient and public involvement group which will inform SCONe as it progresses.

Optometrists’ views on the creation of a large retinal image repository with the goal of creating diagnostic tools using AI methods were gathered in a qualitative study early in SCONe’s development.^18^ This found ‘near universal’ support, with optometrists welcoming anticipated improvements in care with the adoption of such tools.

### Derivation of phenotypes

Our multidisciplinary team prepared a list of codes which identify selected conditions (supplementary material). These are not comprehensive but illustrative of the disease profile of the SCONe cohort and provide an indication of the potential range of diseases for which SCONe could support research.

## Results

### Cohort overview

**Figure 1:**
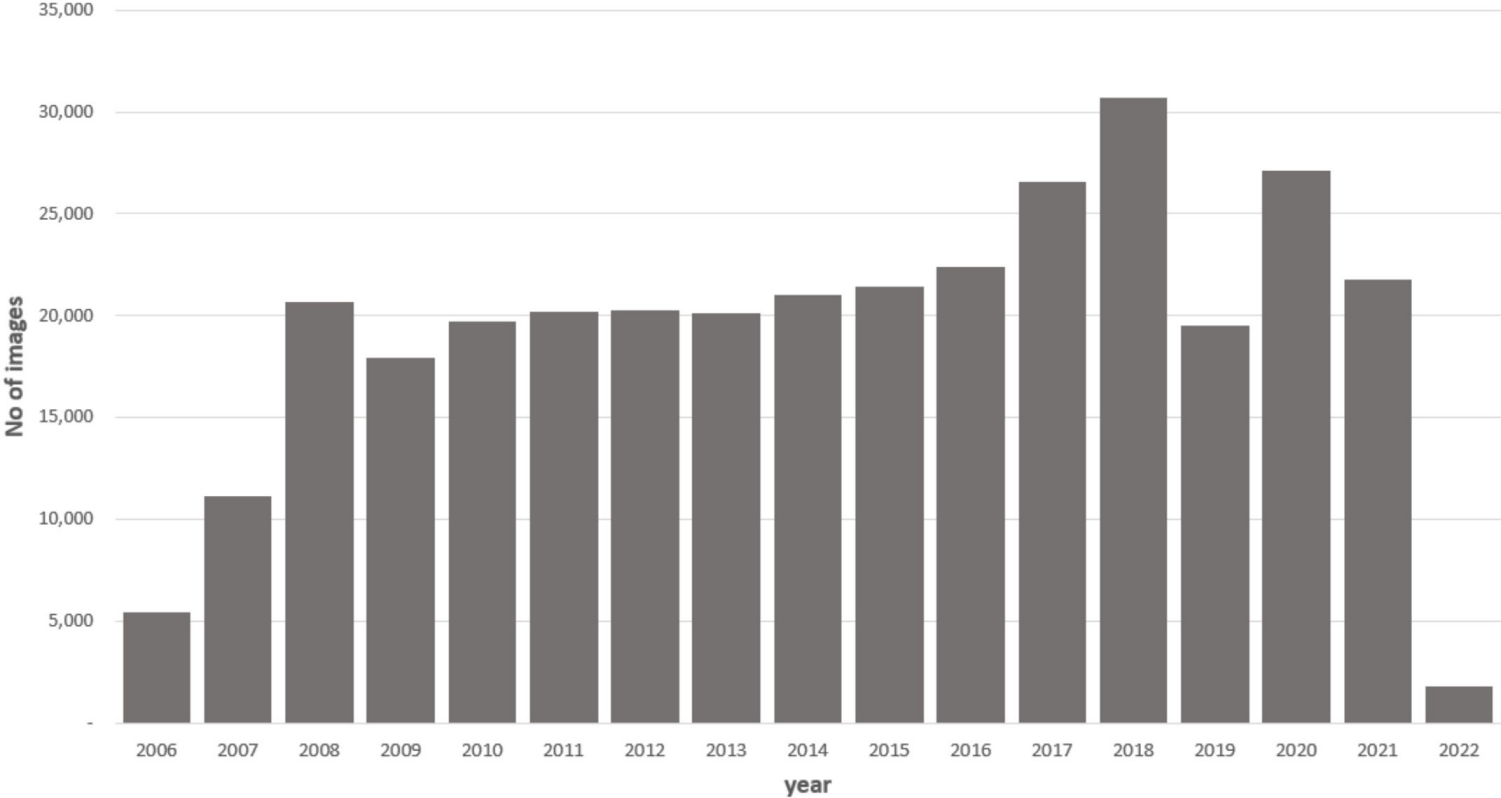
Number of images in the Scottish Collaborative Optometry-Ophthalmology Network e-research (SCONe) cohort by year of capture.

The SCONe retinal image repository contains 366 910 images for 35 704 patients. Initially, images were captured from 11 practices for patients aged 60 and over (on the day of extraction), and in phase 2 (three practices to date), images for patients aged 18+ were copied. Images were captured between January 2006 and September 2023. Patients had between 1 and 227 images each (median 6). The time between the earliest and latest image ranged from 0 to 16.7 years (median 2.7). The number of images captured each year is shown in figure 1.

**Figure 2:**
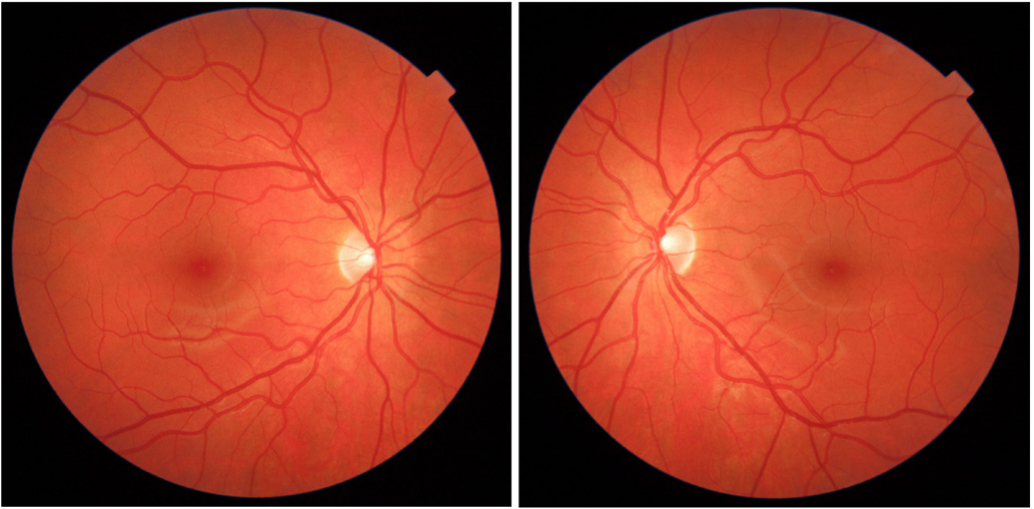
Right and left colour fundus photographs, typical of those included in the Scottish Collaborative Optometry-Ophthalmology Network e-research (SCONe) repository.

SCONe’s fundus photographs (figure 2), typically 45 degrees field of view, were taken by a range of devices, most commonly Topcon (90%; eg, Maestro2, Triton OCT2000; Topcon, Tokyo, Japan), Canon (5%; eg, CRDGI 40D; Canon Medical Systems, Tochigi, Japan) and Nikon (4%; D90, D7100; Nikon, Tokyo, Japan). The most commonly used practice management software was XEYEX (XEYEX, Bellshill, UK), with some using Optix (Optix Software, York, UK) and Acuitas (Ocuco, Dublin, Ireland). Most (96%) images were macula centred, 4% were disc centred. Images and data were collected, including historical information which had been archived from systems no longer in use, sometimes using technical solutions provided by manufacturers. As these systems were not interoperable and the image capture software does not include CHI, a novel intrapractice linkage process was developed and validated, previously described in Tochel *et al*.^19^

**Figure 3:**
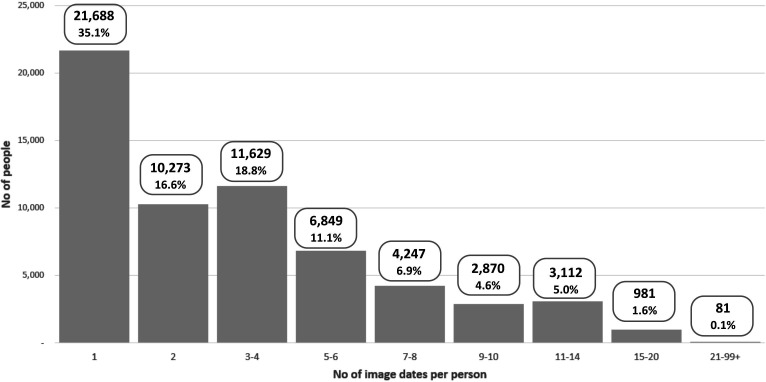
Number of individuals in the current cohort by the number of dates on which Scottish Collaborative Optometry-Ophthalmology Network e-research (SCONe) contains images for them, ranging from 1 to approximately 100.

Figure 3 shows the number of image dates in the cohort per person. This characterises the available longitudinal retinal evidence, as the total number of images may include multiple images taken on the same day.

### Patient demographics

Practice patient details were used by PHS to link each image to CHI (https://www.gov.scot/publications/joined-up-data-better-decisions-guiding-principles-data-linkage/pages/5/). No clinical information was copied from practice, and patient details transferred by the SCONe team were deleted after sharing with PHS for linkage. The following data were all obtained from the coded, linked datasets for patients with one or more retinal images. As some patients had a retinal image but no record in other datasets, there are some missing data. The data are provided with reference to the Scottish population aged 60+ in the 2011 census, as a comparable group.^20^

**Figure 4:**
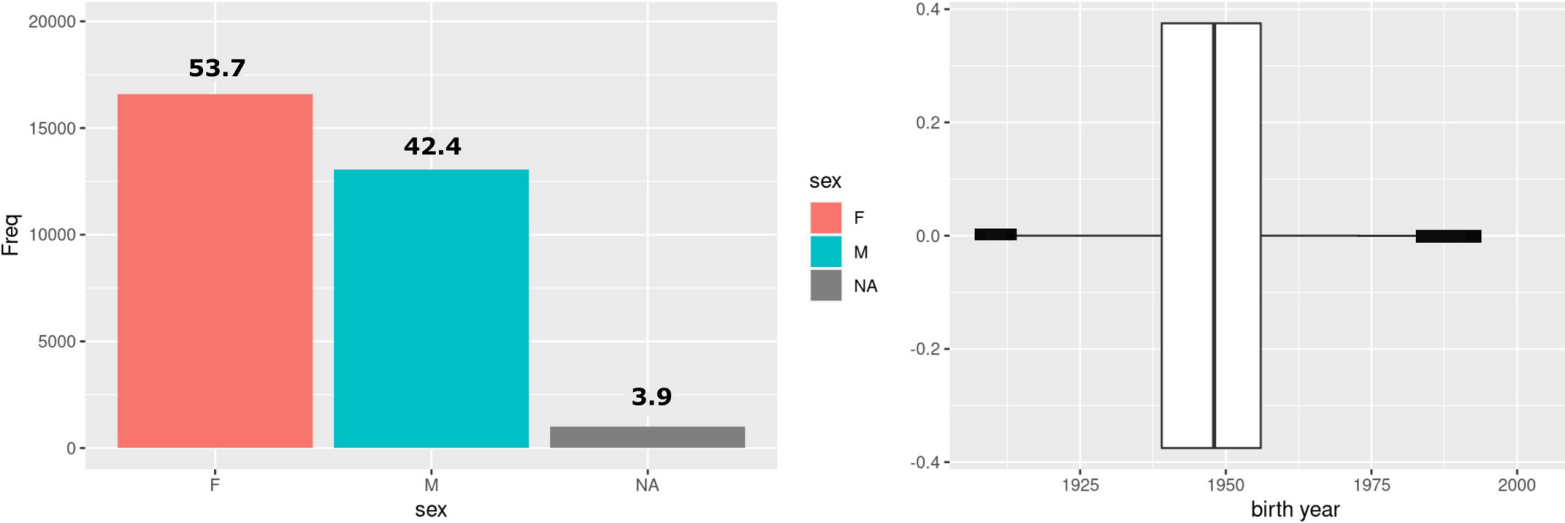
Distribution of sex (left) and birth year (right) for the cohort. Centre line of box plot shows median value, outer edges show IQR, thick line at edges covers outliers.

Figure 4 depicts the sex breakdown of the current SCONe cohort (left), which closely matches the Scottish population (54% female, 42% male), and the distribution of birth year (right) (estimated from the patients’ age at the date of each available healthcare record).

### Sex and year of birth

Table 1 shows the number and percentage of the cohort with information on their ethnic group, compared with the Scottish 60+ population. When the missing data are excluded, it is apparent that the cohort has a slightly higher percentage of people from minority ethnic groups than in the Scottish population.

**Table 1:** Number and percentage of people by ethnic group (collapsed into larger groups to avoid disclosure of small numbers)

	SCONe cohort				Scottish population 60+	
	Including missing		Excluding missing			
	Frequency	%	Frequency	%	Frequency	%
African, Caribbean and black	77	0.2	77	0.3	745	0.1
Asian	348	1.0	348	1.2	8578	0.7
Mixed and multiple	66	0.2	66	0.2	782	0.1
Other	77	0.2	77	0.3	782	0.1
White	27 658	77.5	27 658	98.0	1 215 640	99.1
Unknown, missing and refused	7478	20.9	–	–		
Total	34 327	100	21 087	100	1 226 527	100

SCONe, Scottish Collaborative Optometry-Ophthalmology Network e-research.

**Figure 5:**
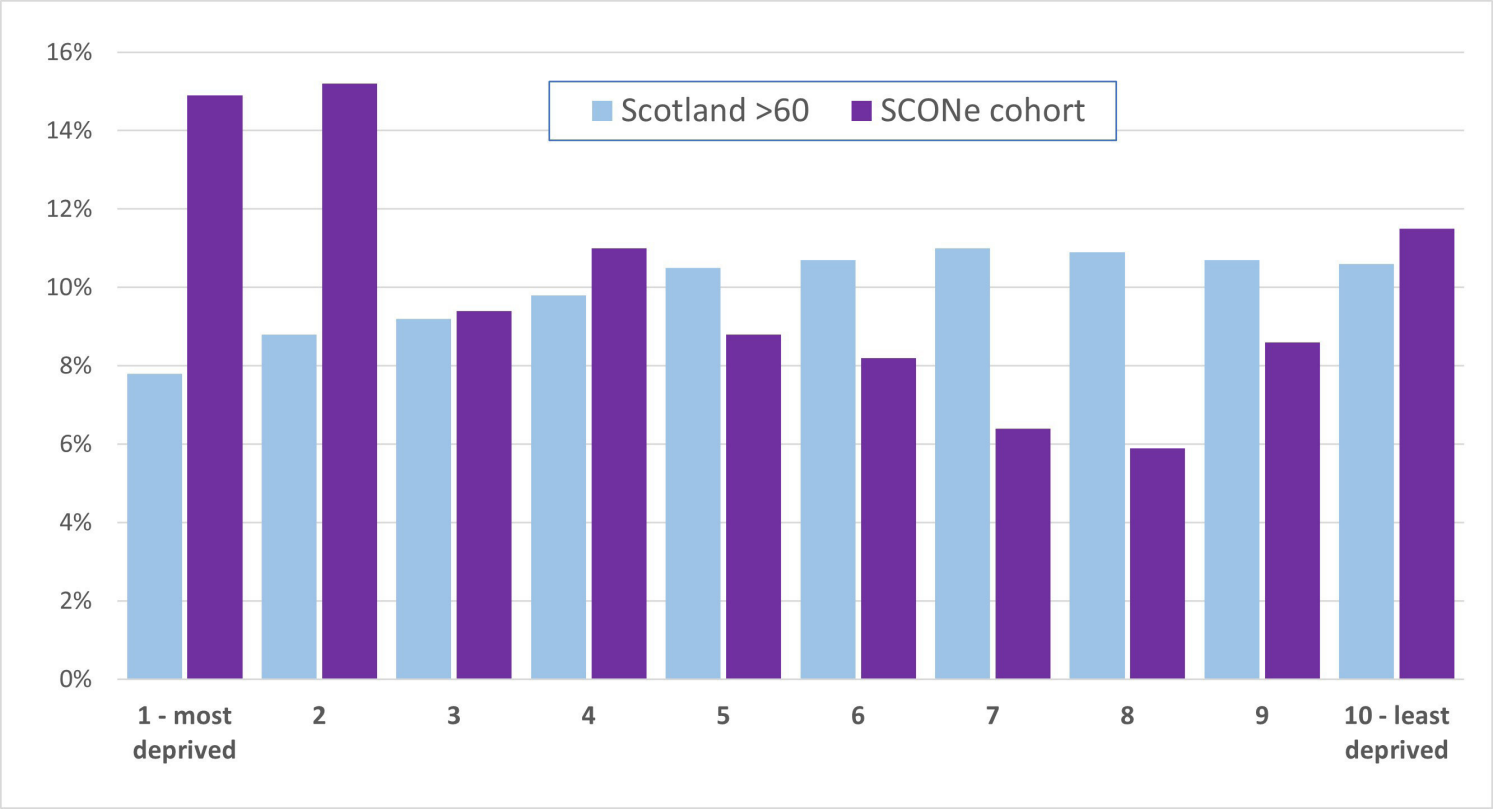
Distribution of Scottish Index of Multiple Deprivation (SIMD) from most deprived (left) to least deprived (right). Blue bars show Scottish population aged 60+ from the 2011 census; purple bars show Scottish Collaborative Optometry-Ophthalmology Network e-research (SCONe) cohort.

Figure 5 shows the distribution of the SIMD in the Scottish 60+ population (blue) and the SCONe cohort (purple), indicating a considerably larger proportion of patients from areas of higher deprivation (towards the left of the graph) in the SCONe cohort.

### Incidence of selected conditions

Using prepared code lists (supplementary material) for the classification systems in use in hospital, death and general ophthalmic data, patients were identified with one or more codes, indicating that a clinician had recorded the condition during a healthcare appointment. For the purpose of this paper, codes from all sources are given the same weight. The earliest date for each condition (regardless of source) was noted per patient and taken as a proxy for their incident diagnosis. This must be interpreted with the caveat that we do not have a complete medical history for every patient. At this stage, code lists for the prescribing data have not yet been developed; this work is in progress and therefore case numbers are underestimated here for many conditions.

Table 2 shows the number of patients in the SCONe cohort with one or more codes for each selected condition and the percentage of the cohort this represents. For comparison, a systematic review of AMD prevalence in Europe suggests that 25.3% of people aged 60+ have early/intermediate AMD and 2.4% have late AMD.^21^ It has been estimated that 3.5% of people aged 40+ have glaucoma.^22^ In the UK, primary open-angle glaucoma prevalence was calculated to increase from 3.7% for people in their 60s to 7.8% for people over 80.^23^ A global systematic review calculated the prevalence of dementia in people aged 60+ as 5–7% in most regions of the world.^24^

**Table 2:** Number of patients and images in the SCONe cohort associated with selected diseases identified through linked data

		Patients		Images		
		Number with 1+ code	Cohort (%)	Total	Number which predates the first code	Number which postdates the first code
Eye	Cataract	25 538	71.5	298 582	80 321	218 261
	Diabetic retinopathy	436	1.2	6375	1974	4280
	Glaucoma	3212	9	48 668	15 393	33 275
	Macular degeneration	2946	8.3	38 051	9888	28 089
	Retinal detachment	370	1	5538	1785	3753
	Other eye conditions	24 178	67.7	279 684	113 558	166 126
Neurodegenerative	Alzheimer’s disease	309	0.9	3109	2766	342
	Cognitive decline	703	2	6537	5417	1120
	Dementia	1630	4.6	14 812	13 547	1265
	Parkinson’s disease	288	0.8	2713	2146	567
Systemic	Diabetes	6691	18.7	79 912	17 697	62 215
	Hypertension and hypertensive heart disease	9217	25.8	100 221	37 993	62 228
	Ischaemic heart disease	6606	18.5	69 691	20 016	49 675
	Pulmonary heart disease	1452	4.1	14 185	9256	4929
	Stroke	2495	7	24 579	14 166	10 413

SCONe, Scottish Collaborative Optometry-Ophthalmology Network e-research.

Subsequently, the ‘incident diagnosis’ date was compared with each patient’s retinal images, and the number of image capture dates which occur before and after this diagnosis was counted, giving an indication of the potential evidence base for disease progression. As expected, eye diseases, or those leading to potential eye complications, have a larger number of images postdating the first disease code than predating it.

It is important to note that as diagnostic and procedural codes in the available classification systems (International Classification of Diseases 10th Revision, GOS or OPCS Classification of Interventions and Procedures v4 (OPCS4)) are not specific to a bodily location (eg, right or left eye), patient image capture date was used as the observational unit. Collectively, the two (or more) images captured on any given date (usually one right eye and one left eye) are considered to contain retinal information relevant to the diagnosis in the SCONe repository.

The table shows that the SCONe cohort already includes many potential cases and controls for a range of diseases, with a large volume of retinal image data to analyse for potential biomarkers, either in advance of disease development or as it progresses.

## Discussion

Scotland is in the unique position globally of having publicly funded, regular, routine eye examinations with retinal imaging for a sizeable proportion of the population (including almost everyone aged 60+) for well over a decade. In 2022/2023, for example, over 60% of 60+ year-olds in Scotland underwent a sight test in primary care optometry.^25^ It is important to protect such health information on behalf of the public, ensuring it is used appropriately and safely in research. Optometrists are required to keep images for 10 years; therefore, many images are already at risk of deletion, with the potential benefit to public health irretrievably lost.

Other retinal image repositories exist (eg, UK Biobank,^26^ Northern Ireland Cohort for the Longitudinal Study of Ageing,^27^ INSIGHT Health Data Research (HDR) UK,^28^ Age-Related Eye Disease Studies^5^), but SCONe benefits from Scotland’s community-acquired retinal images, including years of images prior to the emergence of signs or symptoms of disease across a broad spectrum of the population. This represents rich, longitudinal evidence not commonly available in other retinal image repositories which are mostly compiled through secondary care or disease-specific cohorts, and in some cases consist of photograph scans. There is a great deal of important information to be gleaned from the retina about changes during healthy ageing.

With the backing of the Scottish Government, we will continue to expand SCONe to capture a broad range of common and rare diseases and reflect the country’s population in terms of demographics, geography and socioeconomics. By creating a very large, representative cohort, we will maximise the potential public benefit which can be achieved through research on this invaluable resource. Crucially, participation is voluntary and at the discretion of the optometrists who are the data controllers for their patients’ images.

### Strengths and limitations

SCONe is already larger than most retinal image research repositories and contains individual-level pseudonymous data from multiple linked healthcare datasets. It is stored in a secure location, with access for approved research via PHS. The current SCONe cohort reflects the sex balance in the Scottish 60+ population; it includes a slightly higher proportion of people of black and minority ethnic groups, who are disproportionately affected by eye diseases such as glaucoma and diabetic retinopathy, and a higher proportion of people from lower deprivation indices, offering the potential to explore issues of health inequality. There are hundreds of thousands of images which predate the first diagnosis of many sight-limiting and potentially life-limiting diseases.

Data routinely collected during healthcare appointments inevitably have limitations compared with a bespoke research dataset, for example, diagnostic codes (at a person, rather than an eye level) without detailed clinical findings. The purpose of data collection may be procedural rather than attempting to document a complete medical history; and with extensive longitudinal data, there are inevitably coding changes over time in response to policy updates and new targets around managing specific diseases or patient groups. Our early work is evaluating the impact of this and other issues which may lead to misinterpretation and developing guidance around taking these into account in disease prediction work. Quality assurance has been at the forefront of SCONe data collection from the outset, both in terms of interpreting existing clinical data and analysing retinal images.

### Ongoing developments

We continue to grow the repository (exploring additional dataset linkages such as the national stroke register or brain CT scans) and anticipate that we will have 1 million retinal images within the next 12 months. We will assess the feasibility of collecting optical coherence tomography or ultrawide images; however, devices which capture these are not as widespread as fundus cameras. Therefore, our main focus to achieve a large, representative cohort is on colour fundus photographs.

Inside the National Safe Haven, we are deriving metadata describing common retinal imaging phenotypes (such as vasculature, other anatomical landmarks and disease phenotypes) using automated image feature extraction methodologies and expert manual image grading to add clinically significant details unavailable from routinely collected data. We are also developing analytical methodologies to facilitate meaningful analyses which overcome the challenges inherent in real-world data described above.

### Data sharing

We are conducting curation of the datasets and developing guidance to prepare them for use as a publicly accessible resource in collaboration with PHS. At the moment, access is only possible via partnership with the study sponsors. When the mechanism for broader access is established and ready to accept applications, we will publicise this. In the interim, we will publish metadata on the HDR UK gateway to allow potential users to register their interest.

## Conclusion

The community-based nature of the images underpinning SCONe enables research with a larger health impact than if they were sourced only from secondary care, as they include many healthy and predisease retinas; results will be more generalisable to the wider population. With almost all optometrists across Scotland having access to a fundus camera, SCONe offers the potential to develop a deeply phenotyped retinal image repository ideally placed for algorithm development, novel diagnostics and biomedical discovery. More timely detection of ocular, systemic and neurodegenerative diseases widens the therapeutic window and offers the potential to implement earlier low-risk, cost-effective interventions including lifestyle modification and non-invasive and minimally invasive therapies, and shifts the medical paradigm of care from hospital to community.

## Supplementary material

10.1136/bmjhci-2024-101236 online supplemental material 1

## Data Availability

No data are available.
