# Supplementary material for: SCONe: a community-acquired retinal image repository enabling ocular, cardiovascular and neurodegenerative disease prediction
Source: BMJ Health Care Inform. 2025 May 14;32(1):e101236. doi: 10.1136/bmjhci-2024-101236 (PMC12083377; doi:10.1136/bmjhci-2024-101236)
Supplement: online supplemental material 1 [file bmjhci-32-1-s001.pdf]

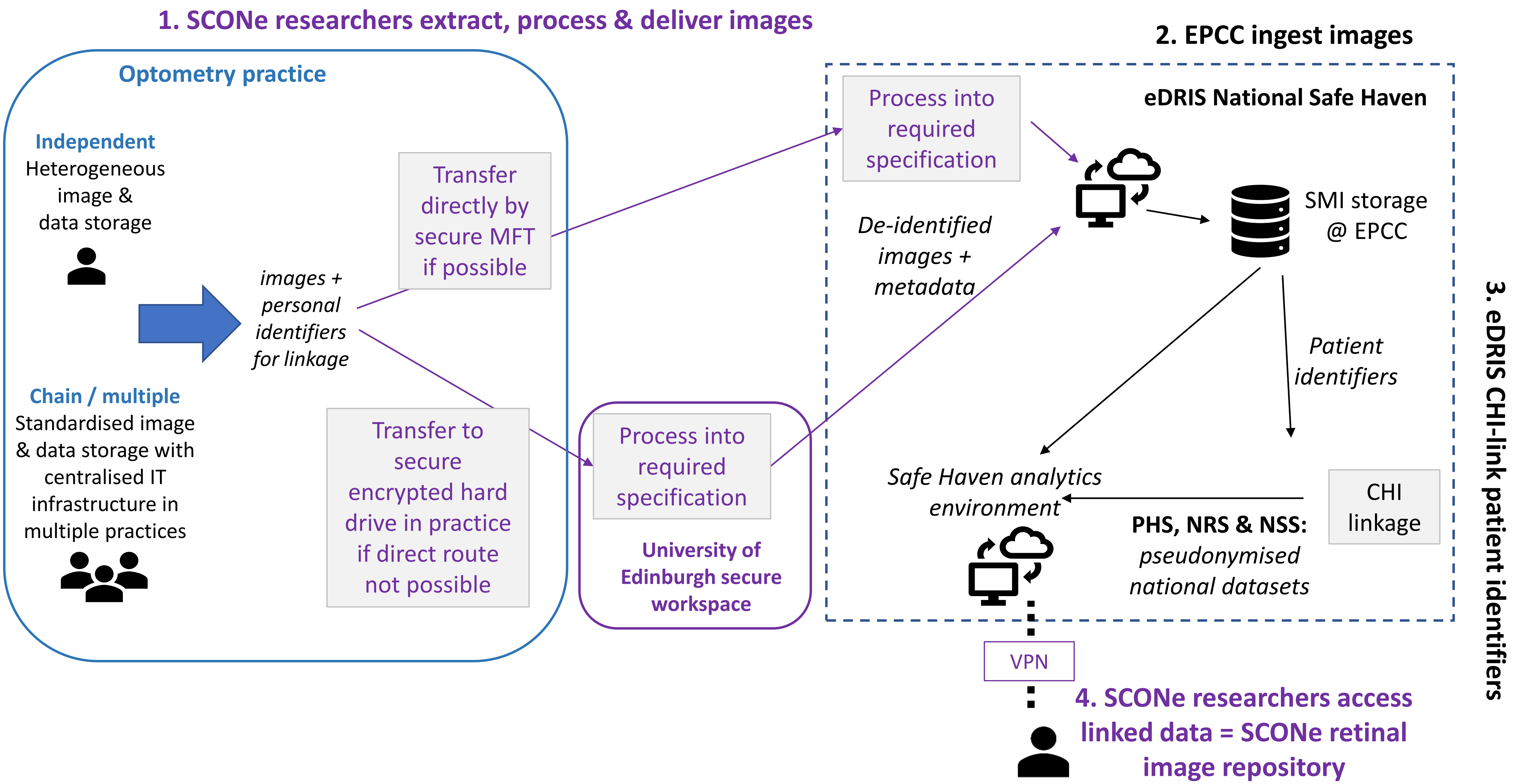
1. SCONe researchers extract, process & deliver images
Optometry practice
Independent
Heterogeneous image & data storage
images + personal identifiers for linkage
Transfer directly by secure MFT if possible
Chain / multiple
Standardised image & data storage with centralised IT infrastructure in multiple practices
Transfer to secure encrypted hard drive in practice if direct route not possible
Process into required specification
University of Edinburgh secure workspace
2. EPCC ingest images
eDRIS National Safe Haven
Process into required specification
De-identified images + metadata
SMI storage @ EPCC
Patient identifiers
CHI linkage
Safe Haven analytics environment
PHS, NRS & NSS:
pseudonymised national datasets
VPN
3. eDRIS CHI-link patient identifiers
4. SCONe researchers access linked data = SCONe retinal image repository

condition label	type of code	source	code	DESCRIPTION from source
alzheimers disease	diagnosis	ICD10	F00	Dementia in Alzheimer disease
alzheimers disease	diagnosis	ICD10	F000	Dementia in Alzheimer disease with early onset
alzheimers disease	diagnosis	ICD10	F001	Dementia in Alzheimer disease with late onset
alzheimers disease	diagnosis	ICD10	F002	Dementia in Alzheimer disease, atypical or mixed type
alzheimers disease	diagnosis	ICD10	F009	Dementia in Alzheimer disease, unspecified
alzheimers disease	diagnosis	ICD10	G30	Alzheimer disease
alzheimers disease	diagnosis	ICD10	G300	Alzheimer disease with early onset
alzheimers disease	diagnosis	ICD10	G301	Alzheimer disease with late onset
alzheimers disease	diagnosis	ICD10	G308	Other Alzheimer disease
alzheimers disease	diagnosis	ICD10	G309	Alzheimer disease, unspecified
brain injury	diagnosis	ICD10	S06	intracranial injury
brain injury	diagnosis	ICD10	S062	diffuse brain injury
brain injury	diagnosis	ICD10	S0620	diffuse brain injury
brain injury	diagnosis	ICD10	S0621	focal brain injury
brain injury	diagnosis	ICD10	S063	focal brain injury
brain injury	diagnosis	ICD10	S0630	focal brain injury
brain injury	diagnosis	ICD10	S0631	focal brain injury
brain injury	diagnosis	ICD10	S067	intracranial injury with prolonged coma
brain injury	diagnosis	ICD10	S0670	intracranial injury with prolonged coma
brain injury	diagnosis	ICD10	S0671	intracranial injury with prolonged coma
brain injury	diagnosis	ICD10	S069	intracranial injury, unspecified
brain injury	diagnosis	ICD10	S0690	intracranial injury, unspecified
brain injury	diagnosis	ICD10	S0691	intracranial injury, unspecified
brain injury	diagnosis	ICD10	T905	sequelae of intracranial injury
cataract	diagnosis	GOS	Cataracts	
cataract	diagnosis	ICD10	H25	Senile cataract
cataract	diagnosis	ICD10	H250	Senile incipient cataract
cataract	diagnosis	ICD10	H251	Senile nuclear cataract
cataract	diagnosis	ICD10	H252	Senile cataract, morgagnian type
cataract	diagnosis	ICD10	H258	Other senile cataract
cataract	diagnosis	ICD10	H259	Senile cataract, unspecified
cataract	diagnosis	ICD10	H26	Other cataract
cataract	diagnosis	ICD10	H260	Infantile, juvenile and presenile cataract
cataract	diagnosis	ICD10	H261	Traumatic cataract
cataract	diagnosis	ICD10	H262	Complicated cataract
cataract	diagnosis	ICD10	H263	Drug-induced cataract
cataract	diagnosis	ICD10	H264	After-cataract
cataract	diagnosis	ICD10	H268	Other specified cataract
cataract	diagnosis	ICD10	H269	Cataract, unspecified
cataract	diagnosis	ICD10	H28	Cataract and other disorders of lens in diseases classified elsewhere
cataract	diagnosis	ICD10	H280	Diabetic cataract
cataract	diagnosis	ICD10	H281	Cataract in other endocrine, nutritional and metabolic diseases
cataract	diagnosis	ICD10	H282	Cataract in other diseases classified elsewhere
cataract	procedure	OPCS4	C71	Extracapsular extraction of lens
cataract	procedure	OPCS4	C71.1	Simple linear extraction of lens
cataract	procedure	OPCS4	C71.2	Phacoemulsification of lens
cataract	procedure	OPCS4	C71.3	Aspiration of lens
cataract	procedure	OPCS4	C71.8	Other specified extracapsular extraction of lens
cataract	procedure	OPCS4	C71.9	Unspecified extracapsular extraction of lens
cataract	procedure	OPCS4	C72	Intracapsular extraction of lens
cataract	procedure	OPCS4	C72.1	Forceps extraction of lens
cataract	procedure	OPCS4	C72.2	Suction extraction of lens
cataract	procedure	OPCS4	C72.3	Cryoextraction of lens
cataract	procedure	OPCS4	C72.8	Other specified intracapsular extraction of lens
cataract	procedure	OPCS4	C72.9	Unspecified intracapsular extraction of lens
cataract	procedure	OPCS4	C74	Other extraction of lens
cataract	procedure	OPCS4	C74.1	Curettage of lens
cataract	procedure	OPCS4	C74.2	Discission of cataract
cataract	procedure	OPCS4	C74.3	Mechanical lensectomy
cataract	procedure	OPCS4	C74.8	Other specified other extraction of lens
cataract	procedure	OPCS4	C74.9	Unspecified other extraction of lens
cataract	procedure	OPCS4	C75	Prosthesis of lens
cataract	procedure	OPCS4	C75.1	Insertion of prosthetic replacement for lens NEC
cataract	procedure	OPCS4	C75.2	Revision of prosthetic replacement for lens
cataract	procedure	OPCS4	C75.3	Removal of prosthetic replacement for lens
cataract	procedure	OPCS4	C75.4	Insertion of prosthetic replacement for lens using suture fixation
cataract	procedure	OPCS4	C75.8	Other specified prosthesis of lens
cataract	procedure	OPCS4	C75.9	Unspecified prosthesis of lens
cognitive decline	diagnosis	ICD10	F067	Mild cognitive disorder
cortical atrophy	diagnosis	ICD10	G310	circumscribed brain atrophy
cortical atrophy	diagnosis	ICD10	G311	senile degeneration of brain, not elsewhere classified
cortical atrophy	diagnosis	ICD10	G318	other specified degenerative diseases of nervous system
cortical atrophy	diagnosis	ICD10	G319	degenerative disease of nervous system, unspecified
dementia	diagnosis	ICD10	F01	Vascular dementia
dementia	diagnosis	ICD10	F010	Vascular dementia of acute onset
dementia	diagnosis	ICD10	F011	Multi-infarct dementia
dementia	diagnosis	ICD10	F012	Subcortical vascular dementia
dementia	diagnosis	ICD10	F013	Mixed cortical and subcortical vascular dementia
dementia	diagnosis	ICD10	F018	Other vascular dementia
dementia	diagnosis	ICD10	F019	Vascular dementia, unspecified
dementia	diagnosis	ICD10	F02	Dementia in other diseases classified elsewhere
dementia	diagnosis	ICD10	F020	Dementia in Pick disease
dementia	diagnosis	ICD10	F021	Dementia in Creutzfeldt-Jakob disease
dementia	diagnosis	ICD10	F022	Dementia in Huntington disease
dementia	diagnosis	ICD10	F023	Dementia in Parkinson disease

dementia	diagnosis	ICD10	F024	Dementia in human immunodeficiency virus [HIV] disease
dementia	diagnosis	ICD10	F028	Dementia in other specified diseases classified elsewhere
dementia	diagnosis	ICD10	F03X	Unspecified dementia
dementia	diagnosis	ICD10	F051	Delirium superimposed on dementia
diabetes	diagnosis	GOS	Diabetes	
diabetes	diagnosis	ICD10	E10	Type 1 diabetes mellitus
diabetes	diagnosis	ICD10	E100	Type 1 diabetes mellitus
diabetes	diagnosis	ICD10	E101	Type 1 diabetes mellitus
diabetes	diagnosis	ICD10	E102	Type 1 diabetes mellitus
diabetes	diagnosis	ICD10	E103	Type 1 diabetes mellitus
diabetes	diagnosis	ICD10	E104	Type 1 diabetes mellitus
diabetes	diagnosis	ICD10	E105	Type 1 diabetes mellitus
diabetes	diagnosis	ICD10	E106	Type 1 diabetes mellitus
diabetes	diagnosis	ICD10	E107	Type 1 diabetes mellitus
diabetes	diagnosis	ICD10	E108	Type 1 diabetes mellitus
diabetes	diagnosis	ICD10	E109	Type 1 diabetes mellitus
diabetes	diagnosis	ICD10	E11	Type 2 diabetes mellitus
diabetes	diagnosis	ICD10	E110	Type 2 diabetes mellitus
diabetes	diagnosis	ICD10	E111	Type 2 diabetes mellitus
diabetes	diagnosis	ICD10	E112	Type 2 diabetes mellitus
diabetes	diagnosis	ICD10	E113	Type 2 diabetes mellitus
diabetes	diagnosis	ICD10	E114	Type 2 diabetes mellitus
diabetes	diagnosis	ICD10	E115	Type 2 diabetes mellitus
diabetes	diagnosis	ICD10	E116	Type 2 diabetes mellitus
diabetes	diagnosis	ICD10	E117	Type 2 diabetes mellitus
diabetes	diagnosis	ICD10	E118	Type 2 diabetes mellitus
diabetes	diagnosis	ICD10	E119	Type 2 diabetes mellitus
diabetes	diagnosis	ICD10	E12	Malnutrition-related diabetes mellitus
diabetes	diagnosis	ICD10	E120	Malnutrition-related diabetes mellitus
diabetes	diagnosis	ICD10	E121	Malnutrition-related diabetes mellitus
diabetes	diagnosis	ICD10	E122	Malnutrition-related diabetes mellitus
diabetes	diagnosis	ICD10	E123	Malnutrition-related diabetes mellitus
diabetes	diagnosis	ICD10	E124	Malnutrition-related diabetes mellitus
diabetes	diagnosis	ICD10	E125	Malnutrition-related diabetes mellitus
diabetes	diagnosis	ICD10	E126	Malnutrition-related diabetes mellitus
diabetes	diagnosis	ICD10	E127	Malnutrition-related diabetes mellitus
diabetes	diagnosis	ICD10	E128	Malnutrition-related diabetes mellitus
diabetes	diagnosis	ICD10	E129	Malnutrition-related diabetes mellitus
diabetes	diagnosis	ICD10	E13	Other specified diabetes mellitus
diabetes	diagnosis	ICD10	E130	Other specified diabetes mellitus
diabetes	diagnosis	ICD10	E131	Other specified diabetes mellitus
diabetes	diagnosis	ICD10	E132	Other specified diabetes mellitus
diabetes	diagnosis	ICD10	E133	Other specified diabetes mellitus
diabetes	diagnosis	ICD10	E134	Other specified diabetes mellitus
diabetes	diagnosis	ICD10	E135	Other specified diabetes mellitus
diabetes	diagnosis	ICD10	E136	Other specified diabetes mellitus
diabetes	diagnosis	ICD10	E137	Other specified diabetes mellitus
diabetes	diagnosis	ICD10	E138	Other specified diabetes mellitus
diabetes	diagnosis	ICD10	E139	Other specified diabetes mellitus
diabetes	diagnosis	ICD10	E14	Unspecified diabetes mellitus
diabetes	diagnosis	ICD10	E140	Unspecified diabetes mellitus
diabetes	diagnosis	ICD10	E141	Unspecified diabetes mellitus
diabetes	diagnosis	ICD10	E142	Unspecified diabetes mellitus
diabetes	diagnosis	ICD10	E143	Unspecified diabetes mellitus
diabetes	diagnosis	ICD10	E144	Unspecified diabetes mellitus
diabetes	diagnosis	ICD10	E145	Unspecified diabetes mellitus
diabetes	diagnosis	ICD10	E146	Unspecified diabetes mellitus
diabetes	diagnosis	ICD10	E147	Unspecified diabetes mellitus
diabetes	diagnosis	ICD10	E148	Unspecified diabetes mellitus
diabetes	diagnosis	ICD10	E149	Unspecified diabetes mellitus
diabetic retinopathy	diagnosis	ICD10	H360	Diabetic retinopathy
diabetic retinopathy	procedure	OPCS4	C82	Destruction of lesion of retina
diabetic retinopathy	procedure	OPCS4	C82.1	Cauterisation of lesion of retina
diabetic retinopathy	procedure	OPCS4	C82.2	Cryotherapy to lesion of retina
diabetic retinopathy	procedure	OPCS4	C82.5	Panretinal laser photocoagulation to lesion of retina
diabetic retinopathy	procedure	OPCS4	C82.6	Laser photocoagulation to lesion of retina NEC
diabetic retinopathy	procedure	OPCS4	C82.8	Other specified destruction of lesion of retina
diabetic retinopathy	procedure	OPCS4	C82.9	Unspecified destruction of lesion of retina
glaucoma	diagnosis	GOS	Glaucoma	
glaucoma	diagnosis	ICD10	H40	Glaucoma
glaucoma	diagnosis	ICD10	H400	Glaucoma suspect
glaucoma	diagnosis	ICD10	H401	Primary open-angle glaucoma
glaucoma	diagnosis	ICD10	H402	Primary angle-closure glaucoma
glaucoma	diagnosis	ICD10	H403	Glaucoma secondary to eye trauma
glaucoma	diagnosis	ICD10	H404	Glaucoma secondary to eye inflammation
glaucoma	diagnosis	ICD10	H405	Glaucoma secondary to other eye disorders
glaucoma	diagnosis	ICD10	H406	Glaucoma secondary to drugs
glaucoma	diagnosis	ICD10	H408	Other glaucoma
glaucoma	diagnosis	ICD10	H409	Glaucoma, unspecified
glaucoma	diagnosis	ICD10	H42	Glaucoma in diseases classified elsewhere
glaucoma	diagnosis	ICD10	H420	Glaucoma in endocrine, nutritional and metabolic diseases
glaucoma	diagnosis	ICD10	H428	Glaucoma in other diseases classified elsewhere
glaucoma	procedure	OPCS4	C52	Excision of sclera
glaucoma	procedure	OPCS4	C52.1	Deep sclerectomy with spacer
glaucoma	procedure	OPCS4	C52.2	Deep sclerectomy without spacer
glaucoma	procedure	OPCS4	C52.8	Other specified excision of sclera

glaucoma	procedure	OPCS4	C52.9	Unspecified excision of sclera
glaucoma	procedure	OPCS4	C59	Excision of iris
glaucoma	procedure	OPCS4	C59.1	Iridocyclectomy
glaucoma	procedure	OPCS4	C59.2	Surgical iridectomy
glaucoma	procedure	OPCS4	C59.8	Other specified excision of iris
glaucoma	procedure	OPCS4	C59.9	Unspecified excision of iris
glaucoma	procedure	OPCS4	C60	Filtering operations on iris
glaucoma	procedure	OPCS4	C60.1	Trabeculectomy
glaucoma	procedure	OPCS4	C60.2	Inclusion of iris
glaucoma	procedure	OPCS4	C60.3	Fixation of iris
glaucoma	procedure	OPCS4	C60.4	Iridoplasty NEC
glaucoma	procedure	OPCS4	C60.5	Insertion of tube into anterior chamber of eye to assist drainage of aqueous humour
glaucoma	procedure	OPCS4	C60.6	Viscocanulostomy
glaucoma	procedure	OPCS4	C60.8	Other specified filtering operations on iris
glaucoma	procedure	OPCS4	C60.9	Unspecified filtering operations on iris
glaucoma	procedure	OPCS4	C61	Other operations on trabecular meshwork of eye
glaucoma	procedure	OPCS4	C61.1	Laser trabeculoplasty
glaucoma	procedure	OPCS4	C61.2	Trabeculotomy
glaucoma	procedure	OPCS4	C61.3	Goniotomy
glaucoma	procedure	OPCS4	C61.4	Goniopuncture
glaucoma	procedure	OPCS4	C61.5	Viscogonioplasty
glaucoma	procedure	OPCS4	C61.8	Other specified other operations on trabecular meshwork of eye
glaucoma	procedure	OPCS4	C61.9	Unspecified other operations on trabecular meshwork of eye
glaucoma	procedure	OPCS4	C62	Incision of iris
glaucoma	procedure	OPCS4	C62.1	Iridosclerotomy
glaucoma	procedure	OPCS4	C62.2	Surgical iridotomy
glaucoma	procedure	OPCS4	C62.3	Laser iridotomy
glaucoma	procedure	OPCS4	C62.4	Correction iridodialysis NEC
glaucoma	procedure	OPCS4	C62.8	Other specified incision of iris
glaucoma	procedure	OPCS4	C62.9	Unspecified incision of iris
glaucoma	procedure	OPCS4	C65	Operations following glaucoma surgery
glaucoma	procedure	OPCS4	C65.1	Needling of bleb
glaucoma	procedure	OPCS4	C65.2	Injection of bleb
glaucoma	procedure	OPCS4	C65.3	Revision of bleb NEC
glaucoma	procedure	OPCS4	C65.4	Removal of releasable suture following glaucoma surgery
glaucoma	procedure	OPCS4	C65.5	Laser suture lysis following glaucoma surgery
glaucoma	procedure	OPCS4	C65.8	Other specified operations following glaucoma surgery
glaucoma	procedure	OPCS4	C65.9	Unspecified operations following glaucoma surgery
hypertensive heart dise	diagnosis	ICD10	I10X	Essential (primary) hypertension
hypertensive heart dise	diagnosis	ICD10	I11	Hypertensive heart disease
hypertensive heart dise	diagnosis	ICD10	I110	Hypertensive heart disease with (congestive) heart failure
hypertensive heart dise	diagnosis	ICD10	I119	Hypertensive heart disease without (congestive) heart failure
hypertensive heart dise	diagnosis	ICD10	I12	Hypertensive renal disease
hypertensive heart dise	diagnosis	ICD10	I120	Hypertensive renal disease with renal failure
hypertensive heart dise	diagnosis	ICD10	I129	Hypertensive renal disease without renal failure
hypertensive heart dise	diagnosis	ICD10	I13	Hypertensive heart and renal disease
hypertensive heart dise	diagnosis	ICD10	I130	Hypertensive heart and renal disease with (congestive) heart failure
hypertensive heart dise	diagnosis	ICD10	I131	Hypertensive heart and renal disease with renal failure
hypertensive heart dise	diagnosis	ICD10	I132	Hypertensive heart and renal disease with both (congestive) heart failure and renal failure
hypertensive heart dise	diagnosis	ICD10	I139	Hypertensive heart and renal disease, unspecified
hypertensive heart dise	diagnosis	ICD10	I15	Secondary hypertension
hypertensive heart dise	diagnosis	ICD10	I150	Renovascular hypertension
hypertensive heart dise	diagnosis	ICD10	I151	Hypertension secondary to other renal disorders
hypertensive heart dise	diagnosis	ICD10	I152	Hypertension secondary to endocrine disorders
hypertensive heart dise	diagnosis	ICD10	I158	Other secondary hypertension
hypertensive heart dise	diagnosis	ICD10	I159	Secondary hypertension, unspecified
ischaemic heart disease	diagnosis	ICD10	I20	Angina pectoris
ischaemic heart disease	diagnosis	ICD10	I200	Unstable angina
ischaemic heart disease	diagnosis	ICD10	I201	Angina pectoris with documented spasm
ischaemic heart disease	diagnosis	ICD10	I208	Other forms of angina pectoris
ischaemic heart disease	diagnosis	ICD10	I209	Angina pectoris, unspecified
ischaemic heart disease	diagnosis	ICD10	I21	Acute myocardial infarction
ischaemic heart disease	diagnosis	ICD10	I210	Acute transmural myocardial infarction of anterior wall
ischaemic heart disease	diagnosis	ICD10	I211	Acute transmural myocardial infarction of inferior wall
ischaemic heart disease	diagnosis	ICD10	I212	Acute transmural myocardial infarction of other sites
ischaemic heart disease	diagnosis	ICD10	I213	Acute transmural myocardial infarction of unspecified site
ischaemic heart disease	diagnosis	ICD10	I214	Acute subendocardial myocardial infarction
ischaemic heart disease	diagnosis	ICD10	I219	Acute myocardial infarction, unspecified
ischaemic heart disease	diagnosis	ICD10	I22	Subsequent myocardial infarction
ischaemic heart disease	diagnosis	ICD10	I220	Subsequent myocardial infarction of anterior wall
ischaemic heart disease	diagnosis	ICD10	I221	Subsequent myocardial infarction of inferior wall
ischaemic heart disease	diagnosis	ICD10	I228	Subsequent myocardial infarction of other sites
ischaemic heart disease	diagnosis	ICD10	I229	Subsequent myocardial infarction of unspecified site
ischaemic heart disease	diagnosis	ICD10	I23	Certain current complications following acute myocardial infarction
ischaemic heart disease	diagnosis	ICD10	I230	Haemopericardium as current complication following acute myocardial infarction
ischaemic heart disease	diagnosis	ICD10	I231	Atrial septal defect as current complication following acute myocardial infarction
ischaemic heart disease	diagnosis	ICD10	I232	Ventricular septal defect as current complication following acute myocardial infarction
ischaemic heart disease	diagnosis	ICD10	I233	Rupture of cardiac wall without haemopericardium as current complication following acute myocardial infarction

ischaemic heart disease diagnosis	ICD10	I234	Rupture of chordae tendineae as current complication following acute myocardial infarction
ischaemic heart disease diagnosis	ICD10	I235	Rupture of papillary muscle as current complication following acute myocardial infarction
ischaemic heart disease diagnosis	ICD10	I236	Thrombosis of atrium, auricular appendage, and ventricle as current complications following acute myocardial infarction
ischaemic heart disease diagnosis	ICD10	I238	Other current complications following acute myocardial infarction
ischaemic heart disease diagnosis	ICD10	I24	Other acute ischaemic heart diseases
ischaemic heart disease diagnosis	ICD10	I240	Coronary thrombosis not resulting in myocardial infarction
ischaemic heart disease diagnosis	ICD10	I241	Dressler syndrome
ischaemic heart disease diagnosis	ICD10	I248	Other forms of acute ischaemic heart disease
ischaemic heart disease diagnosis	ICD10	I249	Acute ischaemic heart disease, unspecified
ischaemic heart disease diagnosis	ICD10	I25	Chronic ischaemic heart disease
ischaemic heart disease diagnosis	ICD10	I250	Atherosclerotic cardiovascular disease, so described
ischaemic heart disease diagnosis	ICD10	I251	Atherosclerotic heart disease
ischaemic heart disease diagnosis	ICD10	I252	Old myocardial infarction
ischaemic heart disease diagnosis	ICD10	I253	Aneurysm of heart
ischaemic heart disease diagnosis	ICD10	I254	Coronary artery aneurysm and dissection
ischaemic heart disease diagnosis	ICD10	I255	Ischaemic cardiomyopathy
ischaemic heart disease diagnosis	ICD10	I256	Silent myocardial ischaemia
ischaemic heart disease diagnosis	ICD10	I258	Other forms of chronic ischaemic heart disease
ischaemic heart disease diagnosis	ICD10	I259	Chronic ischaemic heart disease, unspecified
ischaemic heart disease procedure	OPCS4	K40	Saphenous vein graft replacement of coronary artery
ischaemic heart disease procedure	OPCS4	K40.1	Saphenous vein graft replacement of one coronary artery
ischaemic heart disease procedure	OPCS4	K40.2	Saphenous vein graft replacement of two coronary arteries
ischaemic heart disease procedure	OPCS4	K40.3	Saphenous vein graft replacement of three coronary arteries
ischaemic heart disease procedure	OPCS4	K40.4	Saphenous vein graft replacement of four or more coronary arteries
ischaemic heart disease procedure	OPCS4	K40.8	Other specified saphenous vein graft replacement of coronary artery
ischaemic heart disease procedure	OPCS4	K40.9	Unspecified saphenous vein graft replacement of coronary artery
ischaemic heart disease procedure	OPCS4	K41	Other autograft replacement of coronary artery
ischaemic heart disease procedure	OPCS4	K41.1	Autograft replacement of one coronary artery NEC
ischaemic heart disease procedure	OPCS4	K41.2	Autograft replacement of two coronary arteries NEC
ischaemic heart disease procedure	OPCS4	K41.3	Autograft replacement of three coronary arteries NEC
ischaemic heart disease procedure	OPCS4	K41.4	Autograft replacement of four or more coronary arteries NEC
ischaemic heart disease procedure	OPCS4	K41.8	Other specified other autograft replacement of coronary artery
ischaemic heart disease procedure	OPCS4	K41.9	Unspecified other autograft replacement of coronary artery
ischaemic heart disease procedure	OPCS4	K42	Allograft replacement of coronary artery
ischaemic heart disease procedure	OPCS4	K42.1	Allograft replacement of one coronary artery
ischaemic heart disease procedure	OPCS4	K42.2	Allograft replacement of two coronary arteries
ischaemic heart disease procedure	OPCS4	K42.3	Allograft replacement of three coronary arteries
ischaemic heart disease procedure	OPCS4	K42.4	Allograft replacement of four or more coronary arteries
ischaemic heart disease procedure	OPCS4	K42.8	Other specified allograft replacement of coronary artery
ischaemic heart disease procedure	OPCS4	K42.9	Unspecified allograft replacement of coronary artery
ischaemic heart disease procedure	OPCS4	K43	Prosthetic replacement of coronary artery
ischaemic heart disease procedure	OPCS4	K43.1	Prosthetic replacement of one coronary artery
ischaemic heart disease procedure	OPCS4	K43.2	Prosthetic replacement of two coronary arteries
ischaemic heart disease procedure	OPCS4	K43.3	Prosthetic replacement of three coronary arteries
ischaemic heart disease procedure	OPCS4	K43.4	Prosthetic replacement of four or more coronary arteries
ischaemic heart disease procedure	OPCS4	K43.8	Other specified prosthetic replacement of coronary artery
ischaemic heart disease procedure	OPCS4	K43.9	Unspecified prosthetic replacement of coronary artery
ischaemic heart disease procedure	OPCS4	K44	Other replacement of coronary artery
ischaemic heart disease procedure	OPCS4	K44.1	Replacement of coronary arteries using multiple methods
ischaemic heart disease procedure	OPCS4	K44.2	Revision of replacement of coronary artery
ischaemic heart disease procedure	OPCS4	K44.8	Other specified other replacement of coronary artery
ischaemic heart disease procedure	OPCS4	K44.9	Unspecified other replacement of coronary artery
ischaemic heart disease procedure	OPCS4	K45	Connection of thoracic artery to coronary artery
ischaemic heart disease procedure	OPCS4	K45.1	Double anastomosis of mammary arteries to coronary arteries
ischaemic heart disease procedure	OPCS4	K45.2	Double anastomosis of thoracic arteries to coronary arteries NEC
ischaemic heart disease procedure	OPCS4	K45.3	Anastomosis of mammary artery to left anterior descending coronary artery
ischaemic heart disease procedure	OPCS4	K45.4	Anastomosis of mammary artery to coronary artery NEC
ischaemic heart disease procedure	OPCS4	K45.5	Anastomosis of thoracic artery to coronary artery NEC
ischaemic heart disease procedure	OPCS4	K45.6	Revision of connection of thoracic artery to coronary artery
ischaemic heart disease procedure	OPCS4	K45.8	Other specified connection of thoracic artery to coronary artery
ischaemic heart disease procedure	OPCS4	K45.9	Unspecified connection of thoracic artery to coronary artery
ischaemic heart disease procedure	OPCS4	K46	Other bypass of coronary artery
ischaemic heart disease procedure	OPCS4	K46.1	Double implantation of mammary arteries into heart
ischaemic heart disease procedure	OPCS4	K46.2	Double implantation of thoracic arteries into heart NEC
ischaemic heart disease procedure	OPCS4	K46.3	Implantation of mammary artery into heart NEC
ischaemic heart disease procedure	OPCS4	K46.4	Implantation of thoracic artery into heart NEC
ischaemic heart disease procedure	OPCS4	K46.5	Revision of implantation of thoracic artery into heart
ischaemic heart disease procedure	OPCS4	K46.8	Other specified other bypass of coronary artery
ischaemic heart disease procedure	OPCS4	K46.9	Unspecified other bypass of coronary artery
ischaemic heart disease procedure	OPCS4	K47	Repair of coronary artery
ischaemic heart disease procedure	OPCS4	K47.1	Endarterectomy of coronary artery
ischaemic heart disease procedure	OPCS4	K47.2	Repair of arteriovenous fistula of coronary artery
ischaemic heart disease procedure	OPCS4	K47.3	Repair of aneurysm of coronary artery
ischaemic heart disease procedure	OPCS4	K47.4	Repair of rupture of coronary artery
ischaemic heart disease procedure	OPCS4	K47.5	Repair of arteriovenous malformation of coronary artery
ischaemic heart disease procedure	OPCS4	K47.8	Other specified repair of coronary artery
ischaemic heart disease procedure	OPCS4	K47.9	Unspecified repair of coronary artery
ischaemic heart disease procedure	OPCS4	K48	Other open operations on coronary artery
ischaemic heart disease procedure	OPCS4	K48.1	Transection of muscle bridge of coronary artery
ischaemic heart disease procedure	OPCS4	K48.2	Transposition of coronary artery NEC
ischaemic heart disease procedure	OPCS4	K48.3	Open angioplasty of coronary artery

ischaemic heart disease	procedure	OPCS4	K48.4	Exploration of coronary artery
ischaemic heart disease	procedure	OPCS4	K48.8	Other specified other open operations on coronary artery
ischaemic heart disease	procedure	OPCS4	K48.9	Unspecified other open operations on coronary artery
ischaemic heart disease	procedure	OPCS4	K49	Transluminal balloon angioplasty of coronary artery
ischaemic heart disease	procedure	OPCS4	K49.1	Percutaneous transluminal balloon angioplasty of one coronary artery
ischaemic heart disease	procedure	OPCS4	K49.2	Percutaneous transluminal balloon angioplasty of multiple coronary arteries
ischaemic heart disease	procedure	OPCS4	K49.3	Percutaneous transluminal balloon angioplasty of bypass graft of coronary artery
ischaemic heart disease	procedure	OPCS4	K49.4	Percutaneous transluminal cutting balloon angioplasty of coronary artery
ischaemic heart disease	procedure	OPCS4	K49.8	Other specified transluminal balloon angioplasty of coronary artery
ischaemic heart disease	procedure	OPCS4	K49.9	Unspecified transluminal balloon angioplasty of coronary artery
ischaemic heart disease	procedure	OPCS4	K50	Other therapeutic transluminal operations on coronary artery
ischaemic heart disease	procedure	OPCS4	K50.1	Percutaneous transluminal laser coronary angioplasty
ischaemic heart disease	procedure	OPCS4	K50.2	Percutaneous transluminal coronary thrombolysis using streptokinase
ischaemic heart disease	procedure	OPCS4	K50.3	Percutaneous transluminal injection of therapeutic substance into coronary artery NEC
ischaemic heart disease	procedure	OPCS4	K50.4	Percutaneous transluminal atherectomy of coronary artery
ischaemic heart disease	procedure	OPCS4	K50.8	Other specified other therapeutic transluminal operations on coronary artery
ischaemic heart disease	procedure	OPCS4	K50.9	Unspecified other therapeutic transluminal operations on coronary artery
ischaemic heart disease	procedure	OPCS4	K51	Diagnostic transluminal operations on coronary artery
ischaemic heart disease	procedure	OPCS4	K51.1	Percutaneous transluminal angioscopy
ischaemic heart disease	procedure	OPCS4	K51.2	Intravascular ultrasound of coronary artery
ischaemic heart disease	procedure	OPCS4	K51.8	Other specified diagnostic transluminal operations on coronary artery
ischaemic heart disease	procedure	OPCS4	K51.9	Unspecified diagnostic transluminal operations on coronary artery
ischaemic heart disease	procedure	OPCS4	K75	Percutaneous transluminal balloon angioplasty and insertion of stent into coronary artery
ischaemic heart disease	procedure	OPCS4	K75.1	Percutaneous transluminal balloon angioplasty and insertion of 1-2 drug-eluting stents into coronary artery
ischaemic heart disease	procedure	OPCS4	K75.2	Percutaneous transluminal balloon angioplasty and insertion of 3 or more drug-eluting stents into coronary artery
ischaemic heart disease	procedure	OPCS4	K75.3	Percutaneous transluminal balloon angioplasty and insertion of 1-2 stents into coronary artery
ischaemic heart disease	procedure	OPCS4	K75.4	Percutaneous transluminal balloon angioplasty and insertion of 3 or more stents into coronary artery NEC
ischaemic heart disease	procedure	OPCS4	K75.8	Other specified percutaneous transluminal balloon angioplasty and insertion of stent into coronary artery
ischaemic heart disease	procedure	OPCS4	K75.9	Unspecified percutaneous transluminal balloon angioplasty and insertion of stent into coronary artery
macula problem	diagnosis	GOS	Macula Problems	
macular degeneration	diagnosis	GOS	AMD	
macular degeneration	diagnosis	ICD10	H353	Degeneration of macula and posterior pole
macular degeneration	procedure	OPCS4	C79.4	Injection into vitreous body NEC
macular degeneration	procedure	OPCS4	C88	Destruction of subretinal lesion
macular degeneration	procedure	OPCS4	C88.2	Photodynamic therapy to subretinal lesion
macular degeneration	procedure	OPCS4	C88.8	Other specified destruction of subretinal lesion
macular degeneration	procedure	OPCS4	C88.9	Unspecified destruction of subretinal lesion
multiple sclerosis	diagnosis	ICD10	G35X	multiple sclerosis
other eye conditions	diagnosis	GOS	BVA, CRE, RPM	
other eye conditions	diagnosis	GOS	External Eye disease	
other eye conditions	diagnosis	GOS	Ocular Hypertension	
other eye conditions	diagnosis	GOS	Sight impaired or severely sight impaired	
other eye conditions	diagnosis	GOS	Vitro Retinal Problems	
other eye conditions	diagnosis	ICD10	H04	Disorders of lacrimal system
other eye conditions	diagnosis	ICD10	H040	Dacryoadenitis
other eye conditions	diagnosis	ICD10	H041	Other disorders of lacrimal gland
other eye conditions	diagnosis	ICD10	H042	Epiphora
other eye conditions	diagnosis	ICD10	H043	Acute and unspecified inflammation of lacrimal passages
other eye conditions	diagnosis	ICD10	H044	Chronic inflammation of lacrimal passages
other eye conditions	diagnosis	ICD10	H045	Stenosis and insufficiency of lacrimal passages
other eye conditions	diagnosis	ICD10	H046	Other changes in lacrimal passages
other eye conditions	diagnosis	ICD10	H048	Other disorders of lacrimal system
other eye conditions	diagnosis	ICD10	H049	Disorder of lacrimal system, unspecified
other eye conditions	diagnosis	ICD10	H05	Disorders of orbit
other eye conditions	diagnosis	ICD10	H050	Acute inflammation of orbit
other eye conditions	diagnosis	ICD10	H051	Chronic inflammatory disorders of orbit
other eye conditions	diagnosis	ICD10	H052	Exophthalmic conditions
other eye conditions	diagnosis	ICD10	H053	Deformity of orbit
other eye conditions	diagnosis	ICD10	H054	Enophthalmos
other eye conditions	diagnosis	ICD10	H055	Retained (old) foreign body following penetrating wound of orbit
other eye conditions	diagnosis	ICD10	H058	Other disorders of orbit
other eye conditions	diagnosis	ICD10	H059	Disorder of orbit, unspecified
other eye conditions	diagnosis	ICD10	H06	Disorders of lacrimal system and orbit in diseases classified elsewhere
other eye conditions	diagnosis	ICD10	H060	Disorders of lacrimal system in diseases classified elsewhere
other eye conditions	diagnosis	ICD10	H061	Parasitic infestation of orbit in diseases classified elsewhere
other eye conditions	diagnosis	ICD10	H062	Dysthyroid exophthalmos
other eye conditions	diagnosis	ICD10	H063	Other disorders of orbit in diseases classified elsewhere
other eye conditions	diagnosis	ICD10	H10	Conjunctivitis
other eye conditions	diagnosis	ICD10	H100	Mucopurulent conjunctivitis
other eye conditions	diagnosis	ICD10	H101	Acute atopic conjunctivitis
other eye conditions	diagnosis	ICD10	H102	Other acute conjunctivitis
other eye conditions	diagnosis	ICD10	H103	Acute conjunctivitis, unspecified

other eye conditions	diagnosis	ICD10	H104	Chronic conjunctivitis
other eye conditions	diagnosis	ICD10	H105	Blepharoconjunctivitis
other eye conditions	diagnosis	ICD10	H108	Other conjunctivitis
other eye conditions	diagnosis	ICD10	H109	Conjunctivitis, unspecified
other eye conditions	diagnosis	ICD10	H11	Other disorders of conjunctiva
other eye conditions	diagnosis	ICD10	H110	Pterygium
other eye conditions	diagnosis	ICD10	H111	Conjunctival degenerations and deposits
other eye conditions	diagnosis	ICD10	H112	Conjunctival scars
other eye conditions	diagnosis	ICD10	H113	Conjunctival haemorrhage
other eye conditions	diagnosis	ICD10	H114	Other conjunctival vascular disorders and cysts
other eye conditions	diagnosis	ICD10	H118	Other specified disorders of conjunctiva
other eye conditions	diagnosis	ICD10	H119	Disorder of conjunctiva, unspecified
other eye conditions	diagnosis	ICD10	H13	Disorders of conjunctiva in diseases classified elsewhere
other eye conditions	diagnosis	ICD10	H130	Filarial infection of conjunctiva
other eye conditions	diagnosis	ICD10	H131	Conjunctivitis in infectious and parasitic diseases classified elsewhere
other eye conditions	diagnosis	ICD10	H132	Conjunctivitis in other diseases classified elsewhere
other eye conditions	diagnosis	ICD10	H133	Ocular pemphigoid
other eye conditions	diagnosis	ICD10	H138	Other disorders of conjunctiva in diseases classified elsewhere
other eye conditions	diagnosis	ICD10	H15	Disorders of sclera
other eye conditions	diagnosis	ICD10	H150	Scleritis
other eye conditions	diagnosis	ICD10	H151	Episcleritis
other eye conditions	diagnosis	ICD10	H158	Other disorders of sclera
other eye conditions	diagnosis	ICD10	H159	Disorder of sclera, unspecified
other eye conditions	diagnosis	ICD10	H16	Keratitis
other eye conditions	diagnosis	ICD10	H160	Corneal ulcer
other eye conditions	diagnosis	ICD10	H161	Other superficial keratitis without conjunctivitis
other eye conditions	diagnosis	ICD10	H162	Keratoconjunctivitis
other eye conditions	diagnosis	ICD10	H163	Interstitial and deep keratitis
other eye conditions	diagnosis	ICD10	H164	Corneal neovascularization
other eye conditions	diagnosis	ICD10	H168	Other keratitis
other eye conditions	diagnosis	ICD10	H169	Keratitis, unspecified
other eye conditions	diagnosis	ICD10	H17	Corneal scars and opacities
other eye conditions	diagnosis	ICD10	H170	Adherent leukoma
other eye conditions	diagnosis	ICD10	H171	Other central corneal opacity
other eye conditions	diagnosis	ICD10	H178	Other corneal scars and opacities
other eye conditions	diagnosis	ICD10	H179	Corneal scar and opacity, unspecified
other eye conditions	diagnosis	ICD10	H18	Other disorders of cornea
other eye conditions	diagnosis	ICD10	H180	Corneal pigmentations and deposits
other eye conditions	diagnosis	ICD10	H181	Bullous keratopathy
other eye conditions	diagnosis	ICD10	H182	Other corneal oedema
other eye conditions	diagnosis	ICD10	H183	Changes in corneal membranes
other eye conditions	diagnosis	ICD10	H184	Corneal degeneration
other eye conditions	diagnosis	ICD10	H185	Hereditary corneal dystrophies
other eye conditions	diagnosis	ICD10	H186	Keratoconus
other eye conditions	diagnosis	ICD10	H187	Other corneal deformities
other eye conditions	diagnosis	ICD10	H188	Other specified disorders of cornea
other eye conditions	diagnosis	ICD10	H189	Disorder of cornea, unspecified
other eye conditions	diagnosis	ICD10	H19	Disorders of sclera and cornea in diseases classified elsewhere
other eye conditions	diagnosis	ICD10	H190	Scleritis and episcleritis in diseases classified elsewhere
other eye conditions	diagnosis	ICD10	H191	Herpesviral keratitis and keratoconjunctivitis
other eye conditions	diagnosis	ICD10	H192	Keratitis and keratoconjunctivitis in other infectious and parasitic diseases classified elsewhere
other eye conditions	diagnosis	ICD10	H193	Keratitis and keratoconjunctivitis in other diseases classified elsewhere
other eye conditions	diagnosis	ICD10	H198	Other disorders of sclera and cornea in diseases classified elsewhere
other eye conditions	diagnosis	ICD10	H20	Iridocyclitis
other eye conditions	diagnosis	ICD10	H200	Acute and subacute iridocyclitis
other eye conditions	diagnosis	ICD10	H201	Chronic iridocyclitis
other eye conditions	diagnosis	ICD10	H202	Lens-induced iridocyclitis
other eye conditions	diagnosis	ICD10	H208	Other iridocyclitis
other eye conditions	diagnosis	ICD10	H209	Iridocyclitis, unspecified
other eye conditions	diagnosis	ICD10	H21	Other disorders of iris and ciliary body
other eye conditions	diagnosis	ICD10	H210	Hyphaema
other eye conditions	diagnosis	ICD10	H211	Other vascular disorders of iris and ciliary body
other eye conditions	diagnosis	ICD10	H212	Degeneration of iris and ciliary body
other eye conditions	diagnosis	ICD10	H213	Cyst of iris, ciliary body and anterior chamber
other eye conditions	diagnosis	ICD10	H214	Pupillary membranes
other eye conditions	diagnosis	ICD10	H215	Other adhesions and disruptions of iris and ciliary body
other eye conditions	diagnosis	ICD10	H218	Other specified disorders of iris and ciliary body
other eye conditions	diagnosis	ICD10	H219	Disorder of iris and ciliary body, unspecified
other eye conditions	diagnosis	ICD10	H22	Disorders of iris and ciliary body in diseases classified elsewhere
other eye conditions	diagnosis	ICD10	H220	Iridocyclitis in infectious and parasitic diseases classified elsewhere
other eye conditions	diagnosis	ICD10	H221	Iridocyclitis in other diseases classified elsewhere
other eye conditions	diagnosis	ICD10	H228	Other disorders of iris and ciliary body in diseases classified elsewhere
other eye conditions	diagnosis	ICD10	H27	Other disorders of lens
other eye conditions	diagnosis	ICD10	H270	Aphakia
other eye conditions	diagnosis	ICD10	H271	Dislocation of lens
other eye conditions	diagnosis	ICD10	H278	Other specified disorders of lens
other eye conditions	diagnosis	ICD10	H279	Disorder of lens, unspecified
other eye conditions	diagnosis	ICD10	H288	Other disorders of lens in diseases classified elsewhere
other eye conditions	diagnosis	ICD10	H30	Chorioretinal inflammation
other eye conditions	diagnosis	ICD10	H300	Focal chorioretinal inflammation
other eye conditions	diagnosis	ICD10	H301	Disseminated chorioretinal inflammation
other eye conditions	diagnosis	ICD10	H302	Posterior cyclitis

other eye conditions	diagnosis	ICD10	H308	Other chorioretinal inflammations
other eye conditions	diagnosis	ICD10	H309	Chorioretinal inflammation, unspecified
other eye conditions	diagnosis	ICD10	H31	Other disorders of choroid
other eye conditions	diagnosis	ICD10	H310	Chorioretinal scars
other eye conditions	diagnosis	ICD10	H311	Choroidal degeneration
other eye conditions	diagnosis	ICD10	H312	Hereditary choroidal dystrophy
other eye conditions	diagnosis	ICD10	H313	Choroidal haemorrhage and rupture
other eye conditions	diagnosis	ICD10	H314	Choroidal detachment
other eye conditions	diagnosis	ICD10	H318	Other specified disorders of choroid
other eye conditions	diagnosis	ICD10	H319	Disorder of choroid, unspecified
other eye conditions	diagnosis	ICD10	H32	Chorioretinal disorders in diseases classified elsewhere
other eye conditions	diagnosis	ICD10	H320	Chorioretinal inflammation in infectious and parasitic diseases classified elsewhere
other eye conditions	diagnosis	ICD10	H328	Other chorioretinal disorders in diseases classified elsewhere
other eye conditions	diagnosis	ICD10	H34	Retinal vascular occlusions
other eye conditions	diagnosis	ICD10	H340	Transient retinal artery occlusion
other eye conditions	diagnosis	ICD10	H341	Central retinal artery occlusion
other eye conditions	diagnosis	ICD10	H342	Other retinal artery occlusions
other eye conditions	diagnosis	ICD10	H348	Other retinal vascular occlusions
other eye conditions	diagnosis	ICD10	H349	Retinal vascular occlusion, unspecified
other eye conditions	diagnosis	ICD10	H35	Other retinal disorders
other eye conditions	diagnosis	ICD10	H350	Background retinopathy and retinal vascular changes
other eye conditions	diagnosis	ICD10	H351	Retinopathy of prematurity
other eye conditions	diagnosis	ICD10	H352	Other proliferative retinopathy
other eye conditions	diagnosis	ICD10	H354	Peripheral retinal degeneration
other eye conditions	diagnosis	ICD10	H355	Hereditary retinal dystrophy
other eye conditions	diagnosis	ICD10	H356	Retinal haemorrhage
other eye conditions	diagnosis	ICD10	H357	Separation of retinal layers
other eye conditions	diagnosis	ICD10	H358	Other specified retinal disorders
other eye conditions	diagnosis	ICD10	H359	Retinal disorder, unspecified
other eye conditions	diagnosis	ICD10	H36	Retinal disorders in diseases classified elsewhere
other eye conditions	diagnosis	ICD10	H368	Other retinal disorders in diseases classified elsewhere
other eye conditions	diagnosis	ICD10	H43	Disorders of vitreous body
other eye conditions	diagnosis	ICD10	H430	Vitreous prolapse
other eye conditions	diagnosis	ICD10	H431	Vitreous haemorrhage
other eye conditions	diagnosis	ICD10	H432	Crystalline deposits in vitreous body
other eye conditions	diagnosis	ICD10	H433	Other vitreous opacities
other eye conditions	diagnosis	ICD10	H438	Other disorders of vitreous body
other eye conditions	diagnosis	ICD10	H439	Disorder of vitreous body, unspecified
other eye conditions	diagnosis	ICD10	H44	Disorders of globe
other eye conditions	diagnosis	ICD10	H440	Purulent endophthalmitis
other eye conditions	diagnosis	ICD10	H441	Other endophthalmitis
other eye conditions	diagnosis	ICD10	H442	Degenerative myopia
other eye conditions	diagnosis	ICD10	H443	Other degenerative disorders of globe
other eye conditions	diagnosis	ICD10	H444	Hypotony of eye
other eye conditions	diagnosis	ICD10	H445	Degenerated conditions of globe
other eye conditions	diagnosis	ICD10	H446	Retained (old) intraocular foreign body, magnetic
other eye conditions	diagnosis	ICD10	H447	Retained (old) intraocular foreign body, nonmagnetic
other eye conditions	diagnosis	ICD10	H448	Other disorders of globe
other eye conditions	diagnosis	ICD10	H449	Disorder of globe, unspecified
other eye conditions	diagnosis	ICD10	H45	Disorders of vitreous body and globe in diseases classified elsewhere
other eye conditions	diagnosis	ICD10	H450	Vitreous haemorrhage in diseases classified elsewhere
other eye conditions	diagnosis	ICD10	H451	Endophthalmitis in diseases classified elsewhere
other eye conditions	diagnosis	ICD10	H458	Other disorders of vitreous body and globe in diseases classified elsewhere
other eye conditions	diagnosis	ICD10	H46X	Optic neuritis
other eye conditions	diagnosis	ICD10	H47	Other disorders of optic [2nd] nerve and visual pathways
other eye conditions	diagnosis	ICD10	H470	Disorders of optic nerve, not elsewhere classified
other eye conditions	diagnosis	ICD10	H471	Papilloedema, unspecified
other eye conditions	diagnosis	ICD10	H472	Optic atrophy
other eye conditions	diagnosis	ICD10	H473	Other disorders of optic disc
other eye conditions	diagnosis	ICD10	H474	Disorders of optic chiasm
other eye conditions	diagnosis	ICD10	H475	Disorders of other visual pathways
other eye conditions	diagnosis	ICD10	H476	Disorders of visual cortex
other eye conditions	diagnosis	ICD10	H477	Disorder of visual pathways, unspecified
other eye conditions	diagnosis	ICD10	H48	Disorders of optic [2nd] nerve and visual pathways in diseases classified elsewhere
other eye conditions	diagnosis	ICD10	H480	Optic atrophy in diseases classified elsewhere
other eye conditions	diagnosis	ICD10	H481	Retrobulbar neuritis in diseases classified elsewhere
other eye conditions	diagnosis	ICD10	H488	Other disorders of optic nerve and visual pathways in diseases classified elsewhere
other eye conditions	diagnosis	ICD10	H49	Paralytic strabismus
other eye conditions	diagnosis	ICD10	H490	Third [oculomotor] nerve palsy
other eye conditions	diagnosis	ICD10	H491	Fourth [trochlear] nerve palsy
other eye conditions	diagnosis	ICD10	H492	Sixth [abducent] nerve palsy
other eye conditions	diagnosis	ICD10	H493	Total (external) ophthalmoplegia
other eye conditions	diagnosis	ICD10	H494	Progressive external ophthalmoplegia
other eye conditions	diagnosis	ICD10	H498	Other paralytic strabismus
other eye conditions	diagnosis	ICD10	H499	Paralytic strabismus, unspecified
other eye conditions	diagnosis	ICD10	H50	Other strabismus
other eye conditions	diagnosis	ICD10	H500	Convergent concomitant strabismus
other eye conditions	diagnosis	ICD10	H501	Divergent concomitant strabismus
other eye conditions	diagnosis	ICD10	H502	Vertical strabismus
other eye conditions	diagnosis	ICD10	H503	Intermittent heterotropia
other eye conditions	diagnosis	ICD10	H504	Other and unspecified heterotropia

other eye conditions	diagnosis	ICD10	H505	Heterophoria
other eye conditions	diagnosis	ICD10	H506	Mechanical strabismus
other eye conditions	diagnosis	ICD10	H508	Other specified strabismus
other eye conditions	diagnosis	ICD10	H509	Strabismus, unspecified
other eye conditions	diagnosis	ICD10	H51	Other disorders of binocular movement
other eye conditions	diagnosis	ICD10	H510	Palsy of conjugate gaze
other eye conditions	diagnosis	ICD10	H511	Convergence insufficiency and excess
other eye conditions	diagnosis	ICD10	H512	Internuclear ophthalmoplegia
other eye conditions	diagnosis	ICD10	H518	Other specified disorders of binocular movement
other eye conditions	diagnosis	ICD10	H519	Disorder of binocular movement, unspecified
other eye conditions	diagnosis	ICD10	H52	Disorders of refraction and accommodation
other eye conditions	diagnosis	ICD10	H520	Hypermetropia
other eye conditions	diagnosis	ICD10	H521	Myopia
other eye conditions	diagnosis	ICD10	H522	Astigmatism
other eye conditions	diagnosis	ICD10	H523	Anisometropia and aniseikonia
other eye conditions	diagnosis	ICD10	H524	Presbyopia
other eye conditions	diagnosis	ICD10	H525	Disorders of accommodation
other eye conditions	diagnosis	ICD10	H526	Other disorders of refraction
other eye conditions	diagnosis	ICD10	H527	Disorder of refraction, unspecified
other eye conditions	diagnosis	ICD10	H53	Visual disturbances
other eye conditions	diagnosis	ICD10	H530	Amblyopia ex anopsia
other eye conditions	diagnosis	ICD10	H531	Subjective visual disturbances
other eye conditions	diagnosis	ICD10	H532	Diplopia
other eye conditions	diagnosis	ICD10	H533	Other disorders of binocular vision
other eye conditions	diagnosis	ICD10	H534	Visual field defects
other eye conditions	diagnosis	ICD10	H535	Colour vision deficiencies
other eye conditions	diagnosis	ICD10	H536	Night blindness
other eye conditions	diagnosis	ICD10	H538	Other visual disturbances
other eye conditions	diagnosis	ICD10	H539	Visual disturbance, unspecified
other eye conditions	diagnosis	ICD10	H54	Visual impairment including blindness (binocular or monocular)
other eye conditions	diagnosis	ICD10	H540	Blindness, binocular
other eye conditions	diagnosis	ICD10	H541	Severe visual impairment, binocular
other eye conditions	diagnosis	ICD10	H542	Moderate visual impairment, binocular
other eye conditions	diagnosis	ICD10	H543	Mild or no visual impairment, binocular
other eye conditions	diagnosis	ICD10	H544	Blindness, monocular
other eye conditions	diagnosis	ICD10	H545	Severe visual impairment, monocular
other eye conditions	diagnosis	ICD10	H546	Moderate visual impairment, monocular
other eye conditions	diagnosis	ICD10	H549	Unspecified visual impairment (binocular)
other eye conditions	diagnosis	ICD10	H55X	Nystagmus and other irregular eye movements
other eye conditions	diagnosis	ICD10	H57	Other disorders of eye and adnexa
other eye conditions	diagnosis	ICD10	H570	Anomalies of pupillary function
other eye conditions	diagnosis	ICD10	H571	Ocular pain
other eye conditions	diagnosis	ICD10	H578	Other specified disorders of eye and adnexa
other eye conditions	diagnosis	ICD10	H579	Disorder of eye and adnexa, unspecified
other eye conditions	diagnosis	ICD10	H58	Other disorders of eye and adnexa in diseases classified elsewhere
other eye conditions	diagnosis	ICD10	H580	Anomalies of pupillary function in diseases classified elsewhere
other eye conditions	diagnosis	ICD10	H581	Visual disturbances in diseases classified elsewhere
other eye conditions	diagnosis	ICD10	H588	Other specified disorders of eye and adnexa in diseases classified elsewhere
other eye conditions	diagnosis	ICD10	H59	Postprocedural disorders of eye and adnexa, not elsewhere classified
other eye conditions	diagnosis	ICD10	H590	Keratopathy (bullous aphakic) following cataract surgery
other eye conditions	diagnosis	ICD10	H598	Other postprocedural disorders of eye and adnexa
other eye conditions	diagnosis	ICD10	H599	Postprocedural disorder of eye and adnexa, unspecified
other eye conditions	procedure	OPCS4	C89	Operations on posterior segment of eye
parkinsons	diagnosis	ICD10	F023	Dementia in Parkinson disease
parkinsons	diagnosis	ICD10	G20X	parkinson disease
parkinsons	diagnosis	ICD10	Y467	antiparkinsonism drugs
pulmonary heart diseas	diagnosis	ICD10	I26	Pulmonary embolism
pulmonary heart diseas	diagnosis	ICD10	I260	Pulmonary embolism with mention of acute cor pulmonale
pulmonary heart diseas	diagnosis	ICD10	I269	Pulmonary embolism without mention of acute cor pulmonale
pulmonary heart diseas	diagnosis	ICD10	I27	Other pulmonary heart diseases
pulmonary heart diseas	diagnosis	ICD10	I270	Primary pulmonary hypertension
pulmonary heart diseas	diagnosis	ICD10	I271	Kyphoscoliotic heart disease
pulmonary heart diseas	diagnosis	ICD10	I272	Other secondary pulmonary hypertension
pulmonary heart diseas	diagnosis	ICD10	I278	Other specified pulmonary heart diseases
pulmonary heart diseas	diagnosis	ICD10	I279	Pulmonary heart disease, unspecified
pulmonary heart diseas	diagnosis	ICD10	I28	Other diseases of pulmonary vessels
pulmonary heart diseas	diagnosis	ICD10	I280	Arteriovenous fistula of pulmonary vessels
pulmonary heart diseas	diagnosis	ICD10	I281	Aneurysm of pulmonary artery
pulmonary heart diseas	diagnosis	ICD10	I288	Other specified diseases of pulmonary vessels
pulmonary heart diseas	diagnosis	ICD10	I289	Disease of pulmonary vessels, unspecified
retinal detachment	diagnosis	ICD10	H33	Retinal detachments and breaks
retinal detachment	diagnosis	ICD10	H330	Retinal detachment with retinal break
retinal detachment	diagnosis	ICD10	H331	Retinoschisis and retinal cysts
retinal detachment	diagnosis	ICD10	H332	Serous retinal detachment
retinal detachment	diagnosis	ICD10	H333	Retinal breaks without detachment
retinal detachment	diagnosis	ICD10	H334	Traction detachment of retina
retinal detachment	diagnosis	ICD10	H335	Other retinal detachments
retinal detachment	procedure	OPCS4	C81	Photocoagulation of retina for detachment
retinal detachment	procedure	OPCS4	C81.1	Xenon photocoagulation of retina for detachment
retinal detachment	procedure	OPCS4	C81.2	Laser photocoagulation of retina for detachment
retinal detachment	procedure	OPCS4	C81.8	Other specified photocoagulation of retina for detachment
retinal detachment	procedure	OPCS4	C81.9	Unspecified photocoagulation of retina for detachment
retinal detachment	procedure	OPCS4	C85	Fixation of retina
retinal detachment	procedure	OPCS4	C85.1	Retinopexy using cryotherapy
retinal detachment	procedure	OPCS4	C85.2	Retinopexy using diathermy

retinal detachment	procedure	OPCS4	C85.3	Retinopexy using mechanical tacks
retinal detachment	procedure	OPCS4	C85.4	Retinopexy using tissue adhesive
retinal detachment	procedure	OPCS4	C85.5	Retinopexy NEC
retinal detachment	procedure	OPCS4	C85.8	Other specified fixation of retina
retinal detachment	procedure	OPCS4	C85.9	Unspecified fixation of retina
stroke	diagnosis	ICD10	I60	Subarachnoid haemorrhage
stroke	diagnosis	ICD10	I600	Subarachnoid haemorrhage from carotid siphon and bifurcation
stroke	diagnosis	ICD10	I601	Subarachnoid haemorrhage from middle cerebral artery
stroke	diagnosis	ICD10	I602	Subarachnoid haemorrhage from anterior communicating artery
stroke	diagnosis	ICD10	I603	Subarachnoid haemorrhage from posterior communicating artery
stroke	diagnosis	ICD10	I604	Subarachnoid haemorrhage from basilar artery
stroke	diagnosis	ICD10	I605	Subarachnoid haemorrhage from vertebral artery
stroke	diagnosis	ICD10	I606	Subarachnoid haemorrhage from other intracranial arteries
stroke	diagnosis	ICD10	I607	Subarachnoid haemorrhage from intracranial artery, unspecified
stroke	diagnosis	ICD10	I608	Other subarachnoid haemorrhage
stroke	diagnosis	ICD10	I609	Subarachnoid haemorrhage, unspecified
stroke	diagnosis	ICD10	I61	Intracerebral haemorrhage
stroke	diagnosis	ICD10	I610	Intracerebral haemorrhage in hemisphere, subcortical
stroke	diagnosis	ICD10	I611	Intracerebral haemorrhage in hemisphere, cortical
stroke	diagnosis	ICD10	I612	Intracerebral haemorrhage in hemisphere, unspecified
stroke	diagnosis	ICD10	I613	Intracerebral haemorrhage in brain stem
stroke	diagnosis	ICD10	I614	Intracerebral haemorrhage in cerebellum
stroke	diagnosis	ICD10	I615	Intracerebral haemorrhage, intraventricular
stroke	diagnosis	ICD10	I616	Intracerebral haemorrhage, multiple localized
stroke	diagnosis	ICD10	I618	Other intracerebral haemorrhage
stroke	diagnosis	ICD10	I619	Intracerebral haemorrhage, unspecified
stroke	diagnosis	ICD10	I63	Cerebral infarction
stroke	diagnosis	ICD10	I630	Cerebral infarction due to thrombosis of precerebral arteries
stroke	diagnosis	ICD10	I631	Cerebral infarction due to embolism of precerebral arteries
stroke	diagnosis	ICD10	I632	Cerebral infarction due to unspecified occlusion or stenosis of precerebral arteries
stroke	diagnosis	ICD10	I633	Cerebral infarction due to thrombosis of cerebral arteries
stroke	diagnosis	ICD10	I634	Cerebral infarction due to embolism of cerebral arteries
stroke	diagnosis	ICD10	I635	Cerebral infarction due to unspecified occlusion or stenosis of cerebral arteries
stroke	diagnosis	ICD10	I636	Cerebral infarction due to cerebral venous thrombosis, nonpyogenic
stroke	diagnosis	ICD10	I638	Other cerebral infarction
stroke	diagnosis	ICD10	I639	Cerebral infarction, unspecified
stroke	diagnosis	ICD10	I64X	Stroke, not specified as haemorrhage or infarction
